# Boltzmann Energy-based Image Analysis Demonstrates that Extracellular Domain Size Differences Explain Protein Segregation at Immune Synapses

**DOI:** 10.1371/journal.pcbi.1002076

**Published:** 2011-08-04

**Authors:** Nigel J. Burroughs, Karsten Köhler, Vladimir Miloserdov, Michael L. Dustin, P. Anton van der Merwe, Daniel M. Davis

**Affiliations:** 1 Systems Biology Centre, University of Warwick, Coventry, United Kingdom; 2 Cambridge Institute for Medical Research, Addenbrookes Hospital, Cambridge, United Kingdom; 3 Skirball Institute of Biomolecular Medicine, New York University, New York, New York, United States of America; 4 Sir William Dunn School of Pathology, University of Oxford, Oxford, United Kingdom; 5 Division of Cell and Molecular Biology, Imperial College London, London, United Kingdom; University of British Columbia, Canada

## Abstract

Immune synapses formed by T and NK cells both show segregation of the integrin ICAM1 from other proteins such as CD2 (T cell) or KIR (NK cell). However, the mechanism by which these proteins segregate remains unclear; one key hypothesis is a redistribution based on protein size. Simulations of this mechanism qualitatively reproduce observed segregation patterns, but only in certain parameter regimes. Verifying that these parameter constraints in fact hold has not been possible to date, this requiring a quantitative coupling of theory to experimental data. Here, we address this challenge, developing a new methodology for analysing and quantifying image data and its integration with biophysical models. Specifically we fit a binding kinetics model to 2 colour fluorescence data for cytoskeleton independent synapses (2 and 3D) and test whether the observed inverse correlation between fluorophores conforms to size dependent exclusion, and further, whether patterned states are predicted when model parameters are estimated on individual synapses. All synapses analysed satisfy these conditions demonstrating that the mechanisms of protein redistribution have identifiable signatures in their spatial patterns. We conclude that energy processes implicit in protein size based segregation can drive the patternation observed in individual synapses, at least for the specific examples tested, such that no additional processes need to be invoked. This implies that biophysical processes within the membrane interface have a crucial impact on cell∶cell communication and cell signalling, governing protein interactions and protein aggregation.

## Author Summary

A cell interacts with its environment though the thousands of proteins that are expressed on the cell's surface. A number of these proteins are involved in cell∶cell communication, a complex process where two cells establish a (transient) contact interface forming protein bonds that bridge the interface. In T cells, which are a major component of the immune system, the proteins in this interface are organised into spatial domains with small proteins segregating from large ones, a patternation that is believed to be a crucial step in the recognition of antigens by T cells. The mechanism of this patternation is still unresolved, a mechanism based on maximising the number of bonds by partitioning the interface into regions where short and long proteins can form bonds being the most promising. Here we directly compare image patterns with a mathematical theory of patternation demonstrating that there is a signature of the patternation mechanism in the image data. We developed a framework for quantitative modelling of image data in a biophysical context that can be utilised in a variety of other model validation studies. Using these methods we proved that on these images this theory is sufficient to explain the observed patternation.

## Introduction

**Figure 1:**
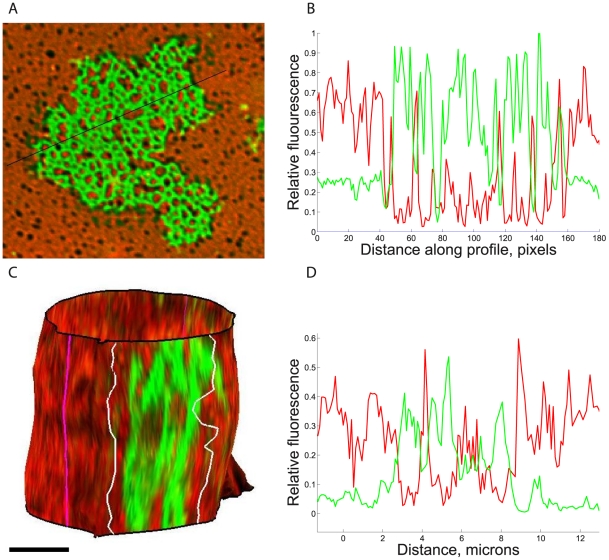
Two colour fluorescence synapse patterns formed by a Jurkat T cell adhering to a lipid bilayer and a NK cell conjugating with a 221 cell. **A**. T cell on a protein-rich supported bilayer loaded with ICAM1 (red) and CD58 (green), from Dustin et al. 1998. **B**. Intensity profile along transect shown in A (black), cell boundary at 5 and 25 microns. **C**. Surface reconstruction of fluorescence on a 221 cell transfected with HLA-Cw6-GFP (green) and ICAM1-Cherry (red). Contact interface limits (and free surface, on rear of cell) are indicated in white (magenta). **D**. Intensity profile along cell surface through contact interface in a mid range z-stack slice; the contact interface extends from approximately 2–9 microns. Bars show 5 microns.

Cell membrane proteins in a number of systems are observed to undergo complex spatial temporal patternation at cell∶cell and cell∶bilayer contact interfaces. Common to these systems is protein segregation according to size, [1], specifically small ligand-receptor pairs (TCR/MHC, KIR/MHC, CD2/CD58, typically 12–15 nm bond length) segregate from proteins with larger extracellular domains (*e.g.* CD45, ICAM1, LFA1, ranging from 18–50 nm, based on structural arguments [1], [2]), Fig. 1. The contact interface within which patternation is observed is called the *immune synapse*, a term that encompasses a variety of patterns. The paradigm was established in the 1990s for T cells interacting with protein-rich supported membrane bilayers [3] and at intercellular contacts [4]. This classic synapse comprises the formation of small MHC clusters that then coalesce, repositioning into a mature bulls-eye structure (pattern coarsening) with ICAM1 positioned in a surrounding annulus [3], [4]. However, many other pattern architectures are reported, including multiple foci in thymocytes [5] and NK cells [6]. Fundamental to these systems is the distinction between segregation of proteins - the partitioning of the surface into domains that are enriched in one or other protein - and aggregation (or pattern coarsening) into a single, normally centralised domain. Experimental evidence now suggests that the latter is an active (ATP-dependent) cytoskeleton driven processes [7]; in particular partitioned supported bilayers reveal a clear centrally orientated force in T cells [8]. Further, this active aggregation is absent in systems where cytoskeletal signalling is inactive or disrupted [9].

Protein segregation can be caused by a variety of processes, including differential protein enrichment in lipid raft microdomains [10], ordering by cytoskeletal processes/actin picket fences [11], [12], specific protein-protein interactions such as tetraspanin-mediated microdomains, or segregation driven by different protein exodomain sizes. The latter process has drawn significant attention from modellers given its (dynamic) self organising property, being distinct from the other mechanisms which are dependent on an ancillary structure or process. Three distinct modelling formulations have been used and all confirm the key result that the coupling of receptor-ligand complexes through the elastic cell membrane can order proteins by size. The resulting (stochastic) spatial patterns qualitatively reproduce observed protein patternation [13]–[16]. The common criterion for instability in these models is that the stretching (or compression) energy to bring the receptor and ligand into sufficiently close proximity to form a bond when the inter membrane separation is different than the natural bond length must be sufficiently high, otherwise uniform protein distributions are thermodynamically preferred [14]. These models also indicate a separation of time scales between size driven domain formation (fast) and pattern coarsening (slow), [14], [17], thereby indicating that T-cell synapse maturation to a bulls eye requires an active (cytoskeletal) mechanism as discussed above. Thus, although the bulls-eye is the minimal energy configuration, the energy surface is insufficiently steep for it to be achieved on realistic time scales by size driven segregation alone. To date, the criterion above for patternation has only been indirectly tested using parameter estimates from the literature, these indicating that patternation by this mechanism is a feasible explanation. However, there has been no direct confirmation that this prediction holds in any experimental system, in part because quantitative comparison of models with spatial image data is extremely difficult. We address this challenge here.

Given the complexity of immunological synapse dynamics, we selected two minimal systems for our analysis. Specifically, we demanded that active cytoskeletal processes are absent. This means that we cannot examine the classic immunological synapse pattern; however there are two well established systems that display segregation in the absence of cytoskeletal processes, Fig. 1. These are, firstly, T cells interacting with a model protein-rich bilayer system containing two fluorescently labelled proteins: CD58 labelled with the green dye FITC, and ICAM1 labelled with the red dye TRITC [9]. Binding can potentially occur between the T cell and the protein-rich bilayer via the T cell surface proteins CD2, which binds CD58, and LFA1, which binds ICAM1. The absence of cytoskeletal activity was hinted at since there is no central aggregation, and demonstrated by inactivation of signalling to the cytoskeleton [9]. Secondly, we examine segregation in live cell-cell conjugates between a Natural Killer (NK) cell, a YTS cell transfected to express the inhibitory receptor KIR2DL1 which binds class I MHC proteins including HLA-Cw6, and a target cell (721.221) transfected to express GFP tagged HLA-Cw6 (HLA-Cw6-GFP) [18] and mCherry tagged ICAM1 (ICAM-Cherry). In this case, binding can potentially occur between the receptor ligand pairs KIR/HLA-Cw6, LFA1/ICAM1 as well as many other receptor/ligand pairs at the surface of the two cells. Since the inhibitory ligand HLA-Cw6 is expressed on the target cells, inhibitory synapses form that are independent of the cytoskeleton [6], [18]. Both these systems show strong segregation and patternation with an enrichment of CD58 (HLA-Cw6 respectively) within the contact interface, Fig. 1. Segregation between the labelled ligands CD58 (HLA-Cw6) and ICAM1 is clearly demonstrated in the line intensity profiles along the surface, Fig. 1B/D, whilst correlation coefficients between the fluorophores in the synapse indicate significant levels of mutual exclusion, values range from −0.39 to −0.69 per synapse (mean −0.49, −0.55 population sd 0.10, 0.08 for bilayer and NK cells respectively, the latter for pixels on the contour). These characteristics of patternation, specifically the mutual exclusion between different sized fluorophores is typical of immune synapses. However, as we demonstrate here, these two systems also show an even greater simplicity than previously thought. Specifically, there is no enrichment of ICAM1 in the interface, Fig. 1, *i.e.* the segregation is between the small receptor-ligand complex (CD2/CD58, KIR/HLA-Cw6 respectively) and the larger unbound ICAM1. The theoretical feasibility of segregation by size in such a system has been previously established [19], whilst it probably represents the minimal system capable of exhibiting self organisation through segregation by size. Thus, these two experimental systems are ideal models for establishing a new framework for quantitative analysis and model comparison.

In this paper we develop a novel energy model that can be used to analyse protein redistribution. We demonstrate that we can extract previously untapped information from two colour fluorescence images. Applying this analysis to observed synapse patterns we are able to quantify the degree of mutual exclusion and specifically test the hypothesis that differences in protein size are sufficient to drive segregation. Thus, by using an energy model parametrised on each individual synapse, we demonstrate for the first time that observed protein segregation patterns in actual synapses can be explained by differences in protein size alone.

## Results

### Explaining fluorescence patterns by ligand binding and protein exclusion

**Figure 2:**
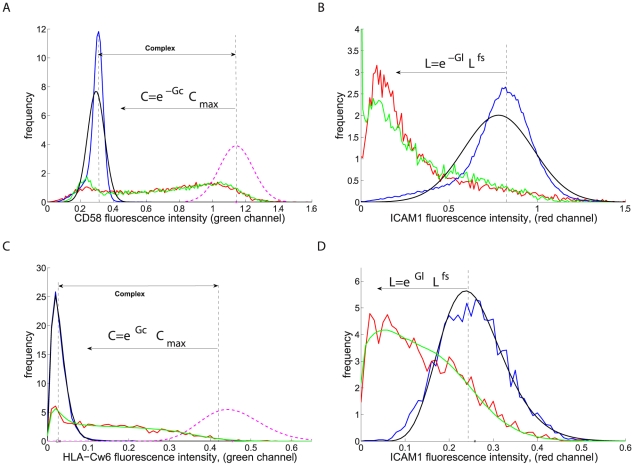
Fluorescence histograms in the contact interface and free surface for typical synapses. Bilayer system showing **A.** CD58, **B.** ICAM1. Inhibitory NK synapse showing **C.** HLA-Cw6 and **D.** ICAM1 (C,D based on pixels on surface contour only). Fluorescence histogram is shown for free surface (blue) with model reconstruction (black) and the contact interface (red), with reconstruction (green). Channels are reconstructed together using a model based on a Gaussian (**A/B**) or Gamma (**C/D**) distribution model and a contact interface potential for the unbound small ligand. The posterior distribution for the optimal binding complex fluorescence is shown (magenta dashed) in **A & C.** Relative energy coordinates 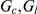 are sketched showing the mapping between fluorescence intensity and energy, shown measured from the (inferred) mean intensity corresponding to optimal binding (A,C) and the mean free surface intensity (B,D) for illustration only.

In both the bilayer and cell conjugate synapses there is an enrichment of the smaller ligand, CD58 (HLA-Cw6), and a concurrent exclusion of the longer ligand, ICAM1 relative to the free surface in distinct regions of the interface, Fig. 2. The remaining part of the interface has fluorescence levels approaching those of the free surface. This indicates that binding is occurring in the interface between the fluorescently tagged ligand CD58 (HLA-Cw6) and its associated receptor CD2 (KIR2DL1), thereby raising the fluorophore concentration above the free surface levels. We do not observe enrichment of ICAM1 in these synapses, Figs. 1 & 2, indicating that negligible binding with LFA1 is occurring. The affinity and avidity of the primary adhesion receptor LFA-1 are subject to signal-dependent upregulation; thus ICAM1 enrichment and contact stabilisation occurs in activating T (and NK) cell synapses through the activation of this pathway [9]. Inhibitory signalling, through KIR for example prevents this inside-out signal and thus prevents ICAM1 enrichment, abrogating adhesion and conjugate formation [20], [21].

The fluorescence intensity histogram, Fig. 2, demonstrates that there is a wide distribution of intensity levels in the interface (compared to the free surface). In particular, the distributions are not bimodal as may have been expected, indicating that the interface environment is highly heterogeneous with regard to the propensity to form complexes. Thus domains are not idealised demarcated entities and show diffuse domain walls with variable levels of fluorophore intensity within the domains. The small ligand shows high levels of enrichment in most pixels in the cell∶bilayer contact whilst a much more diffuse enrichment in NK synapses, Fig. 2; this is because of dominance of the interface by the enriched CD58 (HLA-Cw6) phase. There is also noticeably higher noise in images of intercellular contacts compared with cells stimulated by a protein-rich bilayer, particularly in the mCherry fluorophore, Fig. 1B/D.

The synapse patterns in Fig. 1 are not of the classic T-cell mature synapse variety; there is no centralisation of the small ligand. This is because in both these synapses cytoskeletal processes are not playing a role in reorganisation of the pattern. In the bilayer system, the absence of cytoskeletal transport was confirmed in a truncated CD2 mutant that lacks the cytoskeletal signalling domain [9]. In the inhibitory synapse between YTS:KIR2DL1 and 221:HLA-Cw6-GFP, the organisation of KIR/HLA on a micrometer-scale has been shown to be largely independent of active cytoskeletal rearrangements, at least for these cell types [18].

### Segregation criteria: Model predictions

Mechanisms other than cytoskeletal processes must control protein segregation in these synapses; one possibility is segregation according to protein size [1], [22]. The phenomena works as follows- at the contact interface, bonds form between the small receptor/ligand pair bringing the membranes into close proximity. The intermembrane separation in these regions is likely to be of the order of 12–14 nm, the predicted bond length assuming end on binding [22], [23]. The larger ICAM1, estimated to be 15–20 nm, with dimerisation potentially stiffening the protein, [24], thus experiences an exclusion potential from these regions of close contact. Whether this exclusion is sufficiently strong to give rise to two phases in the interface requires modelling of the system's dynamics and energetics.

Immune synapses have been modelled using a variety of methods. The fundamental division in these approaches is the spatial scale of the modelling. Statistical physics formulations that model individual proteins on a discretised spatial lattice have been simulated (Monte carlo) and analysed, [17], modelling individual receptor-ligand interactions by a rigid square well potential. Thus, binding between facing receptor/ligand pairs occurs only if the membrane separation is within a certain range. These models have previously shown that this system (comprising a long ligand and short receptor-ligand complex) can display patternation [19]. In partial differential equation (PDE) treatments protein concentrations are modelled [13], [14], possibly with noise (stochastic PDEs), and they utilise an effective spring model for receptor/ligand binding in terms of the local membrane separation 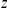, [14],
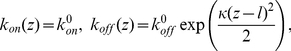
(1)where 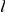 is the natural ‘bond length’ and 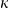 the spring constant (we absorb 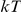 into the spring constants for ease of notation). 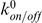 are constants. Here we assume there is no change in the on-rate for simplicity; results are otherwise identical. Since the membrane support is more flexible than the protein, the membrane is essentially the source of this elasticity. A simple model, assuming an infinite elastic sheet gives an effective spring constant of 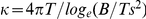 [14], where 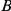, 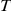 are the membrane rigidity and surface tension respectively and 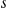 is the radius of the protein in the membrane; this analysis requires 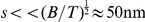 which is satisfied in practice. Thus, the PDE models work at a different scale than the statistical physics models, using an object comprising a receptor-ligand complex and its local supporting membrane as the fundamental unit. This model is only applicable on scales above 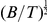, a constraint that is not a problem for light microscopy data of immune synapses. In these models the local membrane separation variable 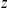 is an average over this length scale. The advantage of these PDE models is their analytically tractability, whilst the fact these two distinctly different modelling formulations give similar predictions indicates that the phenomena is robust to model assumptions.

A PDE model can be derived for this system similar to the two receptor/ligand case [13], [14]. There are 5 coupled differential equations; the molecular species are subject to diffusion and binding, (labels 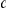 and 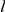 refer to the complex and ICAM1 (long ligand) respectively),
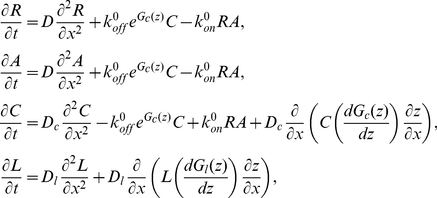
(2)where 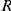, 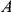 are the (small) receptor and ligand concentrations, 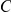 the complex concentration, 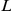 the long ICAM1 concentration, and 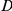, 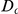 and 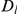 are diffusion constants. We have parametrised the spring energies of Eqn. (1) as,
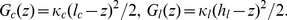
(3)For ICAM1 this comprises an interplay between compression (pushing against the membranes) and the attractive glycocalyx forces. Here 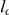, 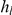 are the natural bond lengths of the CD2/CD58 (KIR/HLA-Cw6) complex and the extracellular domain length of ICAM1 respectively. These elastic forces act on the complex and large ligand introducing a drift potential in Eqns. (2), dragging the complex, resp. large ligand, towards lower energy regions. Finally, the complex and ligand apply force to the membrane(s) introducing spatial heterogeneities in the local separation 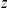 against the restoring elasticity forces, [14],
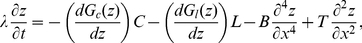
(4)where 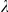 parametrises the response dynamics of the membrane.

This model incorporates the fact that complex formation has a degree of flexibility; the supporting membrane can bend to accommodate different sized protein complexes although this incurs an energy penalty in doing so. It is the balance of these energies that is crucial to patternation, patternation in fact only occurring under certain conditions. To derive these conditions we use a stability analysis following [14]. The analysis considers an initial (spatially) uniform steady state, *i.e.* the inter membrane distance 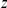 is uniform in the interface and adjusts to establish an equilibrium between bond formation and the cost of exclusion of ICAM1. The protein concentrations determine this balance of energies and thus the equilibrium value(s) of 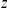. This homogeneous state is then examined for spatial instability, an instability to spatial fluctuations giving rise to a patterned state since the fluctuations will grow in amplitude. This stability analysis (see Supporting Information file Text S1) gives the following condition for the system to exhibit instability (patternation) in spatial mode with wavenumber 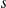 (spatial dependence 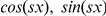),
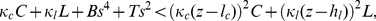
(5)where concentrations (and 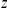) correspond to the uniform steady state. Note that the cell elasticity parameters (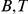) only appear with the wave number 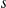 and thus only distinguish relative stability of the spatial modes; it is the spring constants 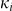 in the PDE formulation that are the key parameters for stability. Condition (5) applies to the steady states for which there are either 1 or 3; again these are a function of the concentrations and the model parameters. Thus, Eqn. (5) determines, firstly for which values of the model parameters can instability occur under any possible conditions (receptor/ligand concentrations, relative area between free surface and cell interface), and secondly, if instability is possible, then for what initial conditions will patternation be observed.

### An energy model for protein relocation

Unfortunately direct fitting of the stochastic analogue of Eqns. (2) to image data is beyond the scope of present methodology. Further, the model implicitly assumes size segregation. The central challenge is thus to model image data using the biophysical principles implicit in the model above in a more general context; *i.e.* with a model that both incorporates essential biophysical features, can be parametrised from the available data whilst capable of producing testable predictions.

Fundamental to an understanding of protein patternation is quantification of the energy demands of protein redistribution and segregation. We thus reparametrise the ICAM1 and complex concentrations in terms of exclusion energies, specifically parametrising in terms of the energy of redistribution relative to a reference (maximum) concentration. For ICAM1 we use the free surface concentration 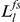 since, in absence of ICAM1 binding, this is the maximum observed concentration in the contact interface, and for CD58 (HLA-Cw6) we define 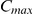 as the optimal (maximal) complex concentration in the interface. By equating chemical potentials this gives, see Fig. 2,
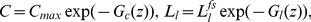
(6)where 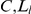 are the local complex and ICAM1 concentration respectively in the contact interface. These relations define the exclusion energies 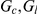 that are dependent on a local environment variable 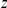. Under an exclusion by size model the local environment parameter 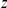 can be identified with the inter membrane separation, achieving a link to the model above. Specifically, this formulation is identical to the PDE model at stationarity, *i.e.* solving for time invariant solutions to Eqns. (2) under general spring energy functions 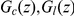, or using the special case of a quadratic local energy dependence Eqns. (3). Thus, 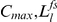 are the concentrations of the complex and ICAM1 under optimal environmental conditions for each species, *i.e.* when 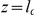 and 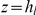 respectively. Since there is an asymmetry between the two species, specifically we have a complex with a small bond length that bridges the two membranes and an unbound ligand with a large extracellular domain, there may be a difference in the effective spring elasticities and thus 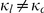.

In general, Eqns. (3) imply that a linear relationship exists between the square roots of the exclusion energies. This is derived by eliminating the unknown (unobserved) intermembrane distance 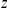, giving,
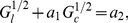
(7)with regression constants 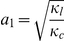, and 
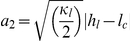
. Since there is mutual exclusion between the species, the positive root for 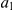 is the physical solution. This relation justifies introduction of the square root energies (SQRE) 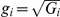 which we use hereafter. The challenge is therefore to use the fluorescence data to estimate the local exclusion energies 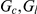, Eqns. (6), and determine if there is evidence of this predicted linear relationship. This presents major difficulties since the fluorescence data is noisy and the complex concentration is not immediately measurable because observed fluorescence is the sum of contributions from the complex and free ligand. To deal with these problems we use a statistical model for the fluorescence intensities.

### A Poisson statistics model for fluorescence intensities

A fluorescence measurement is essentially a counting of the number of contributing fluorophores per pixel. Since fluorophore emission events and concentration fluctuations are independent these events are governed by Poisson statistics; we assume that neighbouring pixels are independent and thus that the dependence incurred through the microscope point spread function (PSF) is removed by deconvolution. The fluorescence 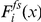 of channel 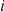 in a free surface (*fs*) pixel 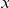 therefore has distribution,
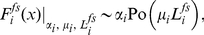
(8)where Po denotes a Poisson distribution. Parameters 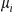 and 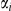 represent the combined emission and detection efficiency, and the fluorescence proportionality constant, respectively. In the free surface, pixels are essentially independent samples informing on the model parameter combinations 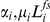. In the contact interface (*ci*), individual pixels are modelled with a local environment dependence through the SQRE, as described above in Eqn. (6), giving for pixel 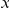,
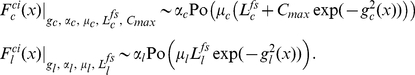
(9)


The bilayer model has 5 global parameter (combinations), namely 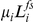, 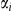, 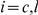, 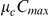, and local parameters 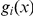 for each pixel in the contact interface. Note that the emission/detection efficiencies 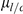 are not estimatable separately from the free ligand concentration.

### Model extensions to 3D

The bilayer patterns are imageable directly, but for patternation on cell surfaces the protein distributions need to be reconstructed from a z-stack (see Text S1). We model each image in the z-stack, extracting regions of free surface and the contact interface along the membrane contour (see Text S1). This requires us to deal with the fact that the cell membrane in each slice (even after deconvolution) has a thickness discernible by light microscopy, typically being wider in the free surface than in the contact interface. This is presumably because the interface suppresses ruffling, whilst optical spreading caused by inexact deconvolution may contribute to this width in all regions. Thus, we extended the model to include an apparent thickness of the membrane under a Gaussian model. By modelling each image we reduce processing artifacts, *e.g.* compared to using a projection which requires distortion of the surface to a plane, whilst also utilising a higher number of pixels in the estimation thereby maximising information extraction. The above model can also be further modified, specifically a Gaussian or Gamma approximation can be used instead of the Poisson distribution above, the latter giving the best fit as it captures the skew in the free surface distribution observed in inter-cell synapses, Fig. 2, cause unknown. In addition we examined a number of model extensions, including inclusion of background autofluorescence and existence of a potential difference for the unbound (small) ligand to diffuse between the contact interface and free surface, this modelling for instance steric or electrostatic effects in the interface. Essentially this discounts the free ligand concentration in the contact interface by factor 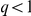, *i.e.* there is a free energy difference of 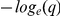.

### Analysis of single cell synapses and model fitting

**Figure 3:**
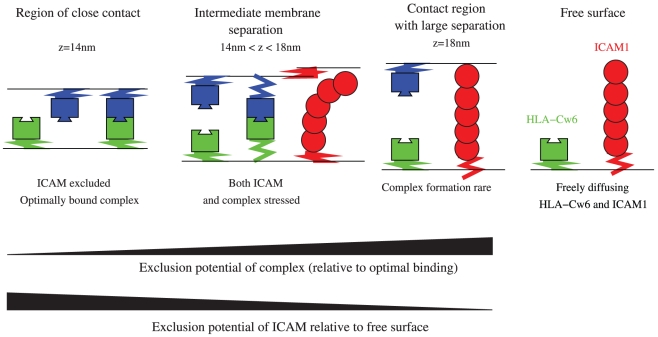
Schematic of energy processes underpinning patternation by size (for the NK synapse). Regions of close contact (from left), intermediate and large separation are shown, illustrating effects on ligand-receptor binding and ICAM1 density. Far right, free surface of target cell with freely diffusing HLA-Cw6 and ICAM1. Below are illustrated the relative exclusion energies (chemical potentials) experienced by the KIR/HLA-Cw6 complex and ICAM1 respectively. Elastic springs are shown, the flexibility in the membrane support allowing complexes to form by pulling the membranes and ICAM1 to locally push the membranes apart.

**Figure 4:**
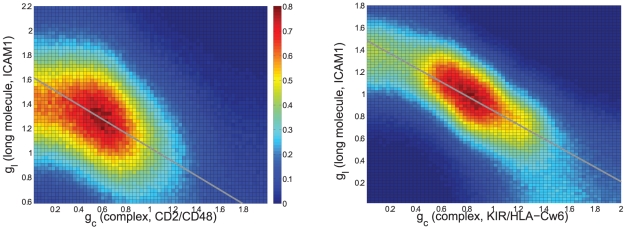
Evidence for a size exclusion mechanism of patternation from exclusion energy quantification. Distribution of inferred square root energies (SQRE) for the two fluorophores shown as a joint probability distribution over the ensemble of contact interface pixels for **A.** a typical bilayer synapse, mean line of regression shown (grey), 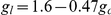, **B.** a typical NK synapse, mean linear regression line (grey), 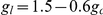. 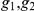 are in units of 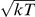.

We fitted the size exclusion model, schematically shown in Fig. 3, to each individual synapse image/z-stack; individual cell fitting allows synapses to be compared and retains key correlations which would otherwise be weakened or lost if synapses are averaged given that synapse patterns are highly variable. We estimate the model parameters for each synapse separately using a Bayesian analysis, (algorithm in Text S1); specifically we estimate concurrently all model parameters by fitting the model to the image data through simulation of the full model posterior distribution. Fluorescence data in the contact interface and a region of the free surface is sufficient to estimate on each channel all model parameters in each synapse (bilayer or cell conjugate); confidence intervals (not shown) for each parameter were reasonable indicating all parameters are estimatable. Our model gives good reproduction of the observed fluorescence histograms, Fig. 2, and further, provides evidence of a strong linear relationship in both the cell∶bilayer and cell∶cell systems, Fig. 4; the first requirement for the segregation by size model. The linear relationship deteriorates at high 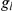 and high 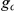 due to degrading signal to noise issues; the fluorescence of the respective species being insufficient to distinguish it from autofluorescence and fluorescence from unbound ligand respectively. This gives rise to the saturation in 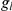 and the spread of the distribution to the right against the barrier, Fig. 4.

**Figure 5:**
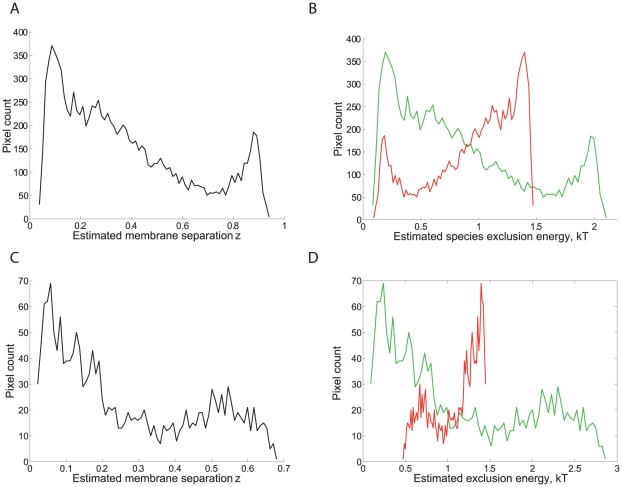
Exclusion energies and membrane height distributions of bilayer and NK contact interfaces. Posterior distributions pooled over pixels in the bilayer of Fig. 1 for **A** distribution of estimated relative membrane height, with 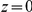 corresponding to CD2–CD58 bond length (12–14 nm), and 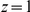 the ICAM1 length (18 nm), **B** exclusion energies of CD2–CD58 complex (green) and ICAM1 (red). Similarly, for the NK synapse of Fig. 1, **C** 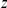, **D** exclusion energies of the KIR/HLA-Cw6 complex (green) and ICAM1 (red).

The analysis was then repeated with the model for size exclusion assumed, specifically Eqn. (7) was imposed thereby estimating the regression parameters 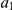 and 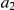. The mean line is shown in Figs. 4. There is no evidence that the gradient of Eqn. (7) is equal to 1 in any of the synapses (posterior probability 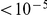) implying a difference in the energy of compression of ICAM1 between the membranes and the stretching of the bond formed by CD2/CD58 or KIR/HLA-Cw6. This asymmetry suggests that accommodation of the long protein ICAM1 in the interface is energetically cheaper than stretching the small bond (or more likely the supporting surface). This makes sense as ICAM1 has additional degrees of freedom since it is not engaged to ligand, and thus presumably able to tilt or possibly even bend, as illustrated in Fig. 3. We reconstructed the hidden variable 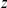 for each pixel, the resulting histogram shows a bimodal distribution for the bilayer interfaces with a mode corresponding to the enriched CD58 phase (close to 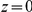), and one corresponding to the competing phase where the CD2-CD58 complex is excluded (close to 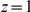), Fig. 5A. For NK cells, the interface is heavily dominated by areas of close contact where HLA-Cw6 is enriched (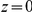); thus bimodality is weaker, Fig. 5C. From an energy perspective, ICAM1 experiences exclusion energies up to 1.5 *kT*, while in certain parts of the interface the complex experiences an exclusion energy of up to 3 *kT*, Fig. 5B/D. In the NK synapse, ICAM1 always experiences an exclusion potential relative to the free surface, minimum 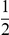 *kT*; this explains why the second mode in Fig. 5C is at 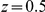 and not nearer 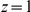.

Our model fit provides estimates of biologically relevant parameters. Typically we obtain an elasticity constant of order 0.1 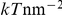 (400 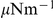) which is consistent with a crude model that approximates the membrane as an elastic sheet and gives an order of magnitude of 40 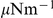 , [14]; cytoskeletal pinning of the membrane is ignored in this estimate suggesting that it is an underestimate. Using mass action, the optimum complex enrichment can be interpreted as 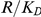, 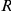 the free receptor concentration in the interface and 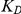 the 2D dissociation constant. Using an order of magnitude estimate of average receptor density on the respective cells (190, 100 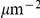) we obtain 2D affinity constant estimates of order 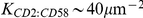, 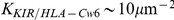. We also find significant evidence of an energy barrier for unbound ligands to enter the contact interface in some of the synapses, the density in the contact interface of CD58, HLA-Cw6-GFP being 37%, 14% lower respectively on average than on the free surface and significantly less than 100% in 1 of 3 bilayers, 4 of 8 NK synapses. This compares to a reduction of 30% experimentally measured using CD48, [25] a non binding ligand.

### Testing instability conditions in single cell synapses

**Figure 6:**
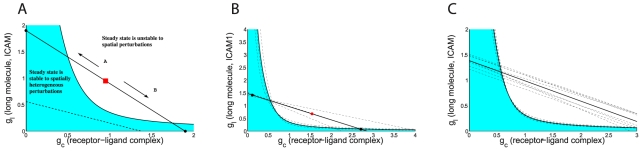
Stability criterion governing patternation. **A.** A point on the SQREs (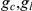) diagram corresponds to an initial spatially uniform configuration prior to patternation, describing the degree of complex formation and ICAM1 exclusion in the interface through Eqns. (3), (6). The line of regression (solid/dashed lines) measured from a synapse image restricts the position of the initial state to this line and also determines the location of the stability curve, (with stable states shaded in blue, unstable, unshaded, and thus forming patterns). The protein concentrations determine the actual homogeneous steady state location, red square. Arrow A: Decreasing the amount of ICAM1 or increasing either the small ligand or receptor concentration moves the steady state towards a higher level of complex formation. Arrow B: vice versa. Small receptor-ligand only and ICAM1 only states are shown, black circles corresponding to 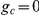 and 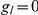 respectively. Two cases are illustrated, solid line, where patternation could be observed within a range of receptor/ligand concentrations, and, dashed, where the homogeneous state is stable at all protein concentrations. The realised steady state and pure molecular species states are shown for the solid line only. **B.** Stability plot for bilayer synapses (individual dashed), mean (solid). Estimated uniform steady states shown, black stable, red unstable for average line. **C.** Stability plots for 8 NK synapses.

The above analysis demonstrates that patternation can be quantitatively parametrised and biologically meaningful parameters determined from experimental images. The next challenge is to address whether extracellular domain size is a primary driver of patternation in these synapses. This requires linking our energy analysis model to the theoretical model of synapse patterning in Eqn. (2). The instability condition, Eqn. (5) for patternation imposes a constraint on the model parameters, a constraint that can be recast in terms of our SQRE coordinates as follows,
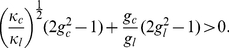
(10)Here, 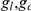 correspond to the uniform steady state SQREs and we have taken 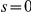 as it is the most unstable mode. The limiting case when the left hand side of Eqn. (10) is equated to zero defines the stability curve. This stability curve is determined by the ratio 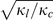, which was in fact estimated directly as the gradient of the regression in Eqn. (7); we thus obtain a direct link between the energy profile analysis of an observed synapse pattern and the dynamic instability criterion which must hold in order that patternation is predicted to occur under the exclusion by size model at those estimated parameter values. Whether the condition on the system parameters in Eqn. (10) holds can be tested simply by observing if there are regions in the 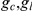 plane where instability is possible, *i.e.* if there is an intersection of the stability curve and the observed line of regression, Fig. 6. As the ligand densities are altered, the equilibrium membrane separation 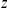 shifts and the uniform steady state defined by 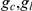 moves along the line of regression, Eqn. (7); thus this line of regression can also be considered the steady state line. If the small or long molecule dominates, *i.e.* 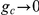 and 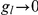 respectively, the system moves out of the region where patternation occurs, Fig. 6. This reproduces the intuitive result that patternation requires an appropriate balance between the concentrations of the long and short ligands.

To test whether this intersection condition holds for our synapses, we estimate the ratio 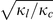 for each synapse. We find that for every synapse there is an intersection of the stability curve and the line of regression, Fig. 6B/C, and thus there exist ligand concentrations where instability is predicted to be observed under the size exclusion mechanism. This leaves the final issue of whether the receptor/ligand concentrations in these synapses are such that patternation would be realised under the size exclusion model. In practice, in the bilayer experiments tuning of the ligand concentrations is performed to find ligand concentrations where patternation (instability) occurs [26], [27], whilst within a population of cells there is sufficient variation of ligand and receptor densities that a small number of patterned synapses are observed for suitable clones in the cell∶cell system. Thus, our proof that the estimated parameters are such that an instability regime exists is already strong confirmation between theory and experiment. This conclusion is also robust to measurement and system noise, the (posterior) probability of no intersection, and therefore patternation not being predicted in any synapse is 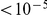.

### Domain seeding rate

If fluorescence is calibrated in terms of molecule density, some additional progress can be made to assess the likelihood of the patterned states being accessible from the initial configuration in the interface. This is an extremely hard question to answer since the contact interface is dynamic, exhibiting spreading against the adjacent surface and undergoing thermal fluctuations. Further, all the necessary parameters or components governing contact dynamics are not known. However, some suggestive results are possible for the bilayer system. Firstly, we can estimate the location of the initial (uniform) state of the synapse prior to patternation for the cell∶bilayer contacts using a previously measured 2D affinity and average contact area [26]. There are 3 uniform states, Fig. 6B, the two extreme stable states correspond to membrane separations close to the CD2-CD58 bond length and the unbound ICAM1 length respectively. The middle state is a compromise configuration of intermediate membrane separation and is unstable to both homogeneous and spatial perturbations. Thus, as observed experimentally, this suggests cells will initially sit on the bilayer without forming CD2-CD58 bonds corresponding to a steady state with 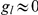 in Fig. 6B, the interface showing no particular enrichment or exclusion of any ligand. In order to patternate, thermal fluctuations are needed to seed a close contact patch where CD2-CD58 bonds can form, thereby leading to exclusion of ICAM1 followed by stabilisation and growth of the patch. Using the analysis and parameters of [28], with an ICAM1 concentration of 500 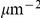, exodomain size 18 nm, patches with a height separation less than 12 nm have an average size of 30 nm, whilst 7% of the surface will be in this close contact regime corresponding to a patch density of the order of 100 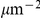. These order of magnitude estimates strongly suggest that seeding of patches is highly likely, and thus the uniform state will eventually patternate.

## Discussion

This is the first report of a thermodynamic analysis of molecule patterning in bilayer and cell surface experimental images. Our new method of fluorescence image analysis uses energy principles to extract novel information from either single or multiple fluorescence data. The method integrates image quantification and biophysical modelling, allowing biologically or physically motivated models to be fitted to image data. Applied to immune synapses, we show that through consideration of the local Boltzmann energy of exclusion that a signature for the segregation process can be identified from two colour fluorescence images in 2 and 3D. This is despite the low levels of signal as indicated by the small energies involved in the patternation, typically only of the order of 1–3 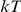, Fig. 5B/D, and thus cellular processes can easily reorganise individual protein molecules. Our analysis shows that when a size exclusion model for patternation is fitted to individual synapses, three levels of model consistency can be analysed. Firstly, the predicted linearity between the square root exclusion energies is clearly evident in both cell∶bilayer and cell∶cell systems, Fig. 4, whilst we observe bimodality in the reconstructed 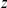 distribution, Fig. 5. Further, the parameter estimates for the bond elasticity extracted from this analysis are consistent with the measured flexibility of the cell membrane in similar cells, whilst our estimates of the order of magnitude for the 2D affinities are reasonable compared to previously measured values, [26]. Previous estimates are an order of magnitude lower at 1 per 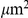, [29], which may indicate that complex formation is suboptimal in synapses, *e.g.* because of a difference in the confinement width between patternated and non patternated interfaces, [30]. However, it is known from theoretical considerations that the 2D affinity is environment dependent [17], with a dependence on receptor/ligand concentrations since binding affects the confinement width through a suppression of fluctuations. These theoretical issues remain to be verified experimentally implying that the concept of a 2D affinity estimate is currently poorly defined. Secondly, we were able to show in all the observed synapses, through estimation of synapse specific model parameters that a protein concentration regime exists when patterned states driven by size differences are predicted to be possible. Finally, on those synapses where the fluorescence intensity was calibrated we obtained order of magnitude estimates of close contact patch sizes and patch frequency suggesting that seeding of patterns from an initial (uniform) membrane separation of 18 nm (ICAM1 length) was likely, *i.e.* the uniform configuration is unstable to thermal fluctuations. We thus conclude that the thermodynamic processes implicit in size exclusion are sufficient to generate the observed patternation and no additional processes need to be invoked. This does not exclude other processes being the cause of, or contributing to segregation; only that as far as has been possible, all predictions of the size exclusion model have been verified. In the NK synapse there are other NK receptor ligands and adhesion molecules that could play a role in the NK synapse patterning; however our results suggest that the main players for synapse organisation are KIR/HLA-Cw6 and ICAM1. Since microscopy required the use of target cells expressing high levels HLA-Cw6 and ICAM1, it is unclear if this also applies to lower expression levels as there are ligand density dependent effects [31].

Our analysis could be improved. Firstly, the PSF also introduces a linear relationship in our 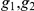 plot. We demonstrate that our results our robust to this effect, see Text S1; however the analysis could be improved through using a Bayesian model selection approach. This would entail incorporating the PSF into the model, and thus removing the deconvolution step; clearly advantageous since deconvolution fixes stochastic noise in the images. Secondly, the analysis could be extended to verify additional model predictions. Specifically, the phase boundaries separating receptor/ligand concentrations where patternation occurs [15] could be ascertained and tested. Our analysis also quantitatively describes the effect of ligand length perturbations [27]; length variation shifts the line of regression in Figs. 4, a prediction that could be directly tested. Extending this analysis to more general synapse systems, including the classic synapse pattern, is the next challenge. An extension to 2 receptor/ligand binding pairs is relatively straight forward; however this model has identifiability problems that will need to be dealt with, *e.g.* through suitable experimental design. The impact of active cytoskeletal processes on our analysis has also not been examined.

In summary, our analysis indicates that segregation in the bilayer and (inhibitory) NK synapse can be explained by size exclusion alone; specifically there is strong evidence for the predicted linearity between 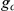, 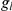 and, using model parameters estimated directly from the observed synapse patterns we find that the instability constraints governing patternation are satisfied in each individual synapse. Our results have important implications. At a methodological level we have demonstrated that two colour fluorescence data contains key information on the mechanisms of protein relocation, information that can be extracted through the techniques developed here. Secondly, our application to the immune synapse shows that at a single cell level biophysical interactions between the cell membrane and embedded proteins lead to self organisation, giving rise to protein segregation, control of ligand binding and aggregation. This ultimately has an impact on signalling [32].

## Materials and Methods

### Bilayer experiments

Experiments were carried out as detailed in [9]. Images were processed for flat field, illumination gradients and background fluorescence was subtracted. The PSF was measured on 100 nm beads and used to deconvolve the image (Richardson-Lucy algorithm). Chromatic aberration was less than a pixel so not corrected. Pixel size is 167 nm. We present results for 3 separate bilayers with 10 synapses.

### NK cell synapses

The HLA-A/B/C negative human EBV-immortalized B-cell line 721.221 [33], was transfected to express HLA-Cw6-GFP and ICAM-Cherry, and cultured as previously described [18], using hygromycin as an additional selection agent for ICAM-Cherry expression. Cells were sorted for high expression levels of both fluorescent proteins using flow cytometry. The ICAM-Cherry plasmid was generated from an ICAM1 with a C-terminal GFP fusion [34] in a pEGFP N-1 vector. The DNA encoding ICAM1 was ligated using the HindIII/BamHI restriction sites into a pcDNA3.1 mCherry vector conferring hygromycin resistance (a kind gift from Marco Purbhoo). 221 cells expressing HLA-Cw6-GFP were transfected by electroporation (Amaxa) according to the manufacturer's instructions and selected with 800 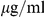 hygromycin (Sigma) for 3 weeks prior to sorting by flow cytometry. The human NK cell line YTS, transfected with the HLA-Cw6 binding inhibitory receptor KIR2DL1 [35], was allowed to form contacts with the target cells for 30 min. A drop of 7 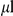 cell suspension in phenol-red-free, HEPES-buffered culture media was mounted between a glass slide and a 22×22 mm coverslip. Imaging was performed at 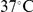 on a confocal laser scanning microscope (TCS SP2, Leica), using a 63× oil immersion objective (1.32 NA), with voxel sizes of 93×93×360 nm. GFP was excited using a laser wavelength of 488 nm, Cherry using 561 nm, and images obtained by sequential excitation. Deconvolution was performed on the basis of the point-spread function determined by imaging fluorescent beads of sub-resolution size. Chromatic aberration was corrected (typically 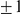 pixel) by maximising the correlation between the channels on the cell of interest; analysis of two colour beads demonstrated that chromatic aberration was not uniform over the image and varied up to a 2 pixel shift in x and y.

### Cell selection (NK synapses)

Cells appropriate for 3D fluorescence reconstruction and modelling had to satisfy a number of criteria, i) have good surface membrane fluorescence in both channels, ii) have low cytosol fluorescence near the membrane, and iii) possess regions of free cell surface (no cell∶cell contact) that were free of ruffling. We used n = 8 synapses in the presented analysis.

### Statistical analysis/computation and model fitting

We developed Markov chain Monte Carlo algorithms to implement a Bayesian inference method for model parameters for both 2D and 3D data. These algorithms simulate the posterior probability of the model parameters given the data through evaluation of the likelihood (see Text S1), from which we can estimate, for instance, their mean values and correlations. We used uniform priors on all parameters, with the interval 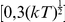 for the SQRE. Convergence was ascertained using a multiple chain protocol [36].

## Supporting Information

Text S1 Text S1 supplies additional information on *Contour tracking*, the algorithm for extracting voxel data from the 3D z stack; an *Instability criterion proof*, *i.e.* proof of Eqns. (5) and (10); a derivation of the *Model likelihood*; a study on the *Effect of PSF on exclusion energy correlation* using the 2D (bilayer) data; and a study of a *Step potential model and PSF linearity*.
(PDF)
