# Supplementary material for: Boltzmann Energy-based Image Analysis Demonstrates that Extracellular Domain Size Differences Explain Protein Segregation at Immune Synapses
Source: PLoS Comput Biol. 2011 Aug 4;7(8):e1002076. doi: 10.1371/journal.pcbi.1002076 (PMC3150282; doi:10.1371/journal.pcbi.1002076)
Supplement: Text S1 — supplies additional information on Contour tracking, the algorithm for extracting voxel data from the 3D z stack; an Instability criterion proof, i.e. proof of Eqns. (5) and (10); a derivation of the Model likelihood; a study on the Effect of PSF on exclusion energy correlation using the 2D (bilayer) data; and a study of a Step potential model and PSF linearity. (PDF) [file pcbi.1002076.s001.pdf]

Text S1.
Supplementary information: Boltzmann energy-based image analysis demonstrates that extracellular domain size differences are sufficient to drive protein segregation at immune synapses

[1*]Nigel J. Burroughs, [2]Karsten Köhler, [1]Vladimir Miloserdov, [3]Michael L. Dustin, [4]P. Anton van der Merwe, [2]Daniel M Davis.

[1]Systems Biology Centre, University of Warwick,
Coventry CV4 7AL. UK.
[2] Division of Cell and Molecular Biology, Imperial College London, South Kensington, London SW7 2AZ, UK.
[3] Skirball Institute of Biomolecular Medicine, NYU, USA.
[4] Sir William Dunn School of Pathology, University of Oxford, South Parks Road, Oxford. OX1 3RE. UK.
[*] Corresponding author. Email: N.J.Burroughs@warwick.ac.uk.

February 17, 2011

Contents

1. Contour tracking
2. Instability criterion proof.
3. Model likelihood
4. Effect of PSF on exclusion energy correlation
5. Step potential model and PSF linearity.

1 Contour tracking.

The 3D z-stack was deconvolved and corrected for chromatic aberation. We tracked the cell membrane contours using a semi-automated algorithm. On a representative z-slice (typically mid-way through the stack) the cell contour was approximated by choosing a number of points along the cell surface. This initial guess was then refined by adding intermediate points and repositioning using a tracking algorithm that estimated the local membrane tangent using the local intensity modes identified from transects across the membrane. Tracking was based on any one of the available fluorophores or a linear combination; in the analysis shown we tended to use a 3:7 to 1:1 weighting of the GFP to cherry images, the GFP tending to be the higher quality image. The final contour had a resolution along the contour of about 1 pixel between points, contour location not being restricted to the image pixelation. This contour was then lifted to neighbouring slices and refined as above.

These contours could be patched together to give a mesh model of the cell surface. Intensity data can be mapped to the contour, thus giving a 3D visualisation of the cell, as Fig. 1C.

Regions of contact interface and free surface were then selected. The s-w coodinates are defined along the contour, s along the centre line, and w orthogonal to s within the image. The $s-w$ coordinates of the centres of each pixel are assigned to that pixel; coordinates for the closest contour centre point are used. In practice the membrane curvature is insufficient to cause ambiguities.

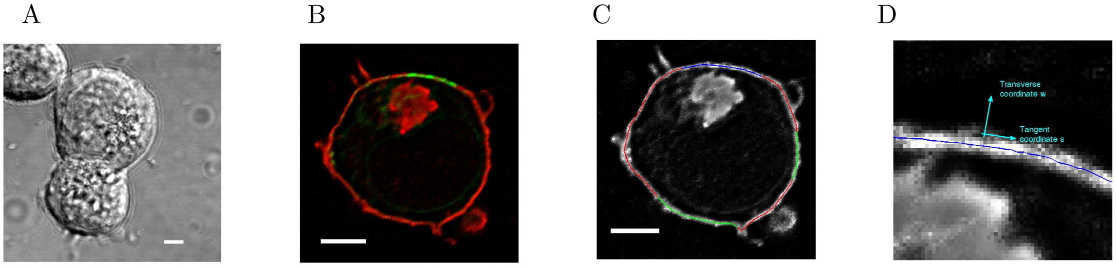

Figure 1. 3D reconstruction of synapses from z-stack images. **A**. Bright field, **B**. processed 2 colour fluorescence of lower cell in A, **C**. contour tracking of cell surface in B shown in red on 1:1 channel weighted image, free surface (green) and contact interface (blue) regions used in analysis. Contour tracking is designed to find an approximate, smooth circumference and thus ignores ruffles. Free surface (FS) and contact interface (CI) regions are selected by hand avoiding highly ruffled regions of surface. Contours and ROIs are then lifted and refined in neighbouring slices. **D**. Local $s-w$ coordinate system shown on a component of free surface. Scale bar is 5 microns.

2 Instability criterion proof.

We prove Eqns. (5) and (10) of the main text for the general potentials $G_c(z), G_l(z)$ under the assumption that $D_l < D$, a reasonable criterion since the long ligand has greater mass. The system inside the contact interface is given by

$$\begin{aligned}
\frac{\partial R}{\partial t} &= D\frac{\partial^2 R}{\partial x^2} + k_{off}^0 e^{G_c(z)} C - k_{on}^0 RA, \\
\frac{\partial A}{\partial t} &= D\frac{\partial^2 A}{\partial x^2} + k_{off}^0 e^{G_c(z)} C - k_{on}^0 RA, \\
\frac{\partial C}{\partial t} &= D_c\frac{\partial^2 C}{\partial x^2} - k_{off}^0 e^{G_c(z)} C + k_{on}^0 RA + D_c \frac{\partial}{\partial x}\left(C\left(\frac{dG_c(z)}{dz}\right)\frac{\partial z}{\partial x}\right), \\
\frac{\partial L}{\partial t} &= D_l\frac{\partial^2 L}{\partial x^2} + D_l \frac{\partial}{\partial x}\left(L\left(\frac{dG_l(z)}{dz}\right)\frac{\partial z}{\partial x}\right), \\
\lambda\frac{\partial z}{\partial t} &= -\left(\frac{dG_c(z)}{dz}\right)C - \left(\frac{dG_l(z)}{dz}\right)L - B\frac{\partial^4 z}{\partial x^4} + T\frac{\partial^2 z}{\partial x^2}.
\end{aligned} \tag{1}$$

For simplicity we assume the receptor R and ligand A have the same diffusion coefficient. Outside the interface $C = 0$, $G_l = 0$ and A, R, L freely diffuse. The system is completed by boundary conditions and matching conditions for the edge of cells, Burroughs & Wulfing, BJ 83 (2002) 1784-96. Note that the difference $R - A$ satisfies the diffusion equation and therefore $R - A$ fluctuations diffuse until $R - A$ is constant. The system is thus simpler than these 5 coupled equations initially suggest.

To proceed with a stability analysis, we firstly determine the steady state conditions. These are given by (prime denotes differentiation with respect to z),

$$G_l'(z)L + G_c'(z)C = 0, \;\; k_{on}AR = Ck_{off}^0 e^{G_c(z)} \tag{2}$$

where A, R, C, L are the uniform (constant) concentrations in the contact interface, and z the uniform separation. To relate these concentrations to the free surface we assume there is no potential difference between the contact interface and free surface giving $A = A^{fs}, R = R^{fs}$, and use the definition of the exclusion potential $G_l(z)$, Eqn. (6), to obtain $L^{fs} = Le^{G_l(z)}$. There are conservation

constraints since the total number of receptor and ligand molecules are conserved assuming the system is closed (*e.g.* an agar plate, cell surface). On solving these conditions there are either 1 or 3 steady states.

The stability analysis proceeds as follows. Consider the system in one of the steady states above. Then compute the growth rates of small fluctuations, assumed sufficiently small that the system can be linearised around the steady state. The spatial modes can then be analysed separately, *i.e.* for modes with spatial depencence $cos(sx), sin(sx)$ we obtain the stability matrix (order $z, A, C, L, R-A$),

$$S = \begin{pmatrix} -\left(G_l''L + G_c''C + Bs^4 + Ts^2\right)\lambda^{-1} & 0 & -G_c'(z)\lambda^{-1} & -G_l'(z)\lambda^{-1} & 0 \\ k_{off}(z)G_c'(z)C & -k_{on}(A+R) - Ds^2 & k_{off}(z) & 0 & -k_{on}A \\ -k_{off}(z)G_c'(z)C - D_cCG_c'(z)s^2 & k_{on}(A+R) & -k_{off}(z) - D_cs^2 & 0 & k_{on}A \\ -s^2D_lLG_l'(z) & 0 & 0 & -D_ls^2 & 0 \\ 0 & 0 & 0 & 0 & -Ds^2 \end{pmatrix} \tag{3}$$

Here $k_{off}(z) = k_{off}^0 e^{G_c(z)}$ and we removed R in favour of $R-A$, which now separates off giving an eigenvalue $-Ds^2$.

The eigenvalues μ are given by $det(S-\mu) = 0$, there being four solutions to find. To prove they are all negative (and thus the steady state stable to spatial perturbations), it is useful to define the determinant,

$$\begin{aligned} f(\mu) &= \begin{vmatrix} -k_{on}(A+R) - Ds^2 - \mu & k_{off}(z) \\ k_{on}(A+R) & -k_{off}(z) - D_cs^2 - \mu \end{vmatrix} \\ &= \left(Ds^2 + \mu\right)\left(D_cs^2 + \mu\right) + k_{off}\left(Ds^2 + \mu\right) + k_{on}(A+R)\left(D_cs^2 + \mu\right) \end{aligned}$$

the central part of S having this form. This determinant has two negative roots $\mu_\pm < -min(D_c, D)s^2$ which follows since $f(\mu)$ is quadratic in μ (with behaviour $f \to \mu^2$ as $\mu \to \pm\infty$), $f(-Ds^2) < 0$ and $f(-D_cs^2) > 0$ if $D_c < D$ or vice versa.

We now get the eigenvalue equation,

$$\begin{aligned} &det(S-\mu) = \\ &\quad -\lambda^{-1}\left(\mu + Ds^2\right)\Big[f(\mu)\left(\mu + D_ls^2\right)\left(\lambda\mu + G_l''L + G_c''C + Bs^4 + Ts^2\right) - f(\mu)D_ls^2L(G_l')^2 - \\ &\qquad G_c'\left(D_ls^2 + \mu\right)\left(k_{off}G_c'C(Ds^2 + \mu) + D_cG_c'Cs^2\left(k_{on}(A+R) + Ds^2 + \mu\right)\right)\Big] = 0 \end{aligned}$$

Thus, the four unknown eigenvalues are given by the crossing points of,

$$\begin{aligned} &\left(\mu + D_ls^2\right)\left(\lambda\mu + G_l''L + G_c''C + Bs^4 + Ts^2\right) - D_ls^2L\left(G_l'\right)^2 = \\ &\quad \frac{1}{f(\mu)}\left(G_c'\right)^2C(D_ls^2 + \mu)\left(k_{off}(Ds^2 + \mu) + D_cs^2\left(k_{on}(A+R) + Ds^2 + \mu\right)\right) \end{aligned} \tag{4}$$

the LHS being a quadratic, the right a function with two poles at $\mu_\pm < 0$ and two zeros, one at $\mu = -D_ls^2$ and the other is negative, satisfying $\mu < -Ds^2$. The RHS is negative at $\mu = -D_ls^2$ (LHS zero), while we assume $D_l < D$ such that this is the zero closest to $\mu = 0$. Further, as $\mu \to \pm\infty$ the LHS $\to \left(k_{off} + D_cs^2\right)\left(G_c'\right)^2 C > 0$. For the case where $\mu_\pm < -D_ls^2$ there are always 3 negative crossings below $\mu = -Ds^2$, Fig. S2; the fourth can be positive depending on if $LHS\big|_{\mu=0} < RHS\big|_{\mu=0}$. Thus, there is a positive eigenvalue if and only if $LHS\big|_{\mu=0} < RHS\big|_{\mu=0}$. Similarly, if $\mu_- < -D_ls^2 < \mu_+$ we obtain 3 negative crossings below μ_+; the fourth crossing is positive under the same conditions. There are no other cases as $D_l < D$ and at least one of $\mu_\pm$ is more negative than $-Ds^2$. The condition $LHS\big|_{\mu=0} < RHS\big|_{\mu=0}$ gives the relation for an instability to heterogeneous perturbations as, ($s \neq 0$)

$$G_l''L + G_c''C + Bs^4 + Ts^2 < L\left(G_l'\right)^2 + C\left(G_c'\right)^2. \tag{5}$$

Clearly, $s \to 0$ is the most unstable mode, ie if it is stable then all modes are stable. This is the state with infinite wavelength; in a closed system the most unstable state will have wavenumber $s = \frac{2\pi}{d}$, d the longest length in the system. In practice $s = 0$ is a good approximation.

This analysis applies for general functional forms for $G_{c/l}$. Using the spring model for the energies we thus obtain Eqn. (5) from Eqn. (S5). Finally, by substituting for $g_l^2 = \frac{\kappa_l}{2}(z-h_l)^2, g_c^2 = \frac{\kappa_c}{2}(z-l_c)^2$ and using the steady state condition Eqn. (S2) we obtain Eqn. (10).

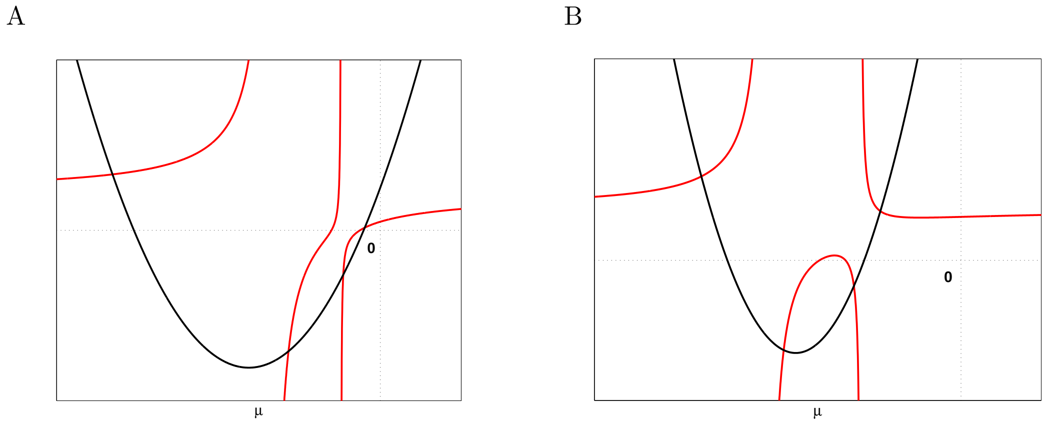

Figure 2. Stability criteria as crossing of curves analysis. Two cases illustrating typical crossings for **A.** with $\mu_\pm < -D_l s^2$, **B.** $\mu_- < -D_l s^2 < \mu_+$. Note the LHS has a zero at $\mu = -D_l s^2$ (most positive zero) whilst the poles are at $\mu_\pm$. The LHS (black), RHS (red) of Eqn. (S4) are shown.

3 Model likelihood.

Here we give the model likelihood for the 3D NK cell flourescence model. This comprises the fluorescence model Eqns. (8),(9) and the model for the variation of fluorescence transverse to the membrane. The global parameters are $\theta_1 = \{\alpha_1, \mu_1, C_1^{max}, L_1^{fs}, q\}$ and $\theta_2 = \{\alpha_2, \mu_2, L_2^{fs}\}$ and local parameters the SQRE $g_i(s)$ and the width parameter $\tau_{fs/ci,i}(s)$, for each position along the contour (both functions of the coordinate s). We use a Gaussian model for fluorescence decay away from the contour centre, precisions $\tau_{fs}(s), \tau_{ci}(s)$, ie allowing for membrane width characteristics to be dependent on the nature of the surface (contact interface or free surface).

The likelihood of the NK (3D) model for separate channel inference is (slice k)

$$\begin{aligned} L_g^k &= p(\tau_{fs,1})p(\tau_{ci,1}) \left(\prod_{x \in fs} p(F_g(x)|\tau_{fs,1}(s_x), \theta_1) \right) \left(\prod_{x \in ci} p(F_g(x)|g_1(w_x), \tau_{ci,1}(s_x), \theta_1) \right) \\ L_r^k &= p(\tau_{fs,2})p(\tau_{ci,2}) \left(\prod_{x \in fs} p(F_r(x)|\tau_{fs,2}(s_x), \theta_2) \right) \left(\prod_{x \in ci} p(F_r(x)|g_2(w_x), \tau_{ci,2}(s_x), \theta_2) \right) \end{aligned} \quad (6)$$

where the respective terms are the model for variation of the membrane width precisions $\tau_{fs/ci,i}(s)$ along the contour, fluorescence at each free surface contour pixel x and fluorescence at each contact interface contour pixel x, the latter conditional on the local environment (local potentials g_i, distance w_x from contour centre). Modelled pixels are close to the contour, typically +/-10 from the contour centre. Here, s, w are the contour coordinates as Fig. S1D. We bin pixels along the contour in segments for the coordinate s; typically a bin is 1 pixel wide. The generalisation to multiple components of free surface and interface is obvious. The likelihood is the product over slices $L_g = \prod_k L_g^k$ etc. For joint inference, $L = \prod_k L_g^k L_r^k$, with $g_i(w)$ constrained by the linear relation Eqn. (7). For the 2D analysis, the slice partitioning and membrane width variables are absent.

We describe the model using the Gaussian approximation to the Poisson model discussed in the text (matching mean and variance). This gives

$$p(F(x)|g_1(w),\tau_1(s),\theta_1) =$$

$$\prod_{x\in fs} \frac{\exp\left(-\frac{(F_1(x)-\alpha_1\mu_1 L_1^{fs} e^{-\frac{1}{2}\tau_{1,fs}(s)w^2})^2}{2\alpha_1^2\mu_1 L_1^{fs} e^{-\tau_{1,fs}(s)w^2/2}}\right)}{\sqrt{2\pi\alpha_1^2\mu_1 L_1^{fs} e^{-\frac{1}{2}\tau_{1,fs}(s)w^2}}} \prod_{x\in ci} \frac{\exp\left(-\frac{\left(F_1(x)-\alpha_1\mu_1\left(C_1^{max}e^{-g_1^2(s)}+qL_1^{fs}\right)e^{-\frac{1}{2}\tau_{1,ci}(s)w^2}\right)^2}{2\alpha_1^2\mu_1\left(C_1^{max}e^{-g_1^2(s)}+qL_1^{fs}\right)e^{-\frac{1}{2}\tau_{1,ci}(s)w^2}}\right)}{\sqrt{2\pi\alpha_1^2\mu_1\left(C_1^{max}e^{-g_1^2(s)}+qL_1^{fs}\right)e^{-\frac{1}{2}\tau_{1,ci}(s)w^2}}},$$

$$p(F(x)|g_2(w),\tau_2(s),\theta_2) =$$

$$\prod_{x\in fs} \frac{\exp\left(-\frac{(F_2(x)-\alpha_2\mu_2 L_2^{fs} e^{-\frac{1}{2}\tau_{2,fs}(s)w^2})^2}{2\alpha_2^2\mu_2 L_2^{fs} e^{-\frac{1}{2}\tau_{2,fs}(s)w^2}}\right)}{\sqrt{2\pi\alpha_2^2\mu_2 L_2^{fs} e^{-\frac{1}{2}\tau_{2,fs}(s)w^2}}} \prod_{x\in ci} \frac{\exp\left(-\frac{(F_2(x)-\alpha_2\mu_2 L_2^{fs} e^{-g_2^2(s)-\frac{1}{2}\tau_{2,ci}(s)w^2})^2}{2\alpha_2^2\mu_2 L_2^{fs} e^{-g_2^2(s)-\frac{1}{2}\tau_{2,ci}(s)w^2}}\right)}{\sqrt{2\pi\alpha_2^2\mu_2 L_2^{fs} e^{-g_2^2(s)-\frac{1}{2}\tau_{2,ci}(s)w^2}}}.$$

where $s = s_x, w = w_x$ are the s-w coordinates of pixel x. Here the products are over all pixels in the free surface and contact interface components of the contour respectively. The Gamma model is similar except the pdf is replaced with that for a Gamma distribution with mean and variance matching the Gaussian above.

For the membrane width, we constrain variation along the membrane by using a random walk model, inferred autoregression parameter d (global, identical across slices and free-surface, contact interface). For each component (free surface, contact interface within each slice) we have, suppressing τ subscripts,

$$p(\boldsymbol{\tau}|d) = \left(p(\tau(s_1))\frac{d^{n/2}}{(2\pi)^{n/2}}\prod_{k=2}^{K}\exp\left(-d\frac{(\tau(s_k)-\tau(s_{k-1}))^2}{2}\right)\right), \quad (7)$$

where K is the number of s-bins in the membrane component, s_i is the s-coordinate of bin i. For the first bin we use $\tau(s_1) \sim Uniform[0,1]$.

A variety of methods can be used to estimate the parameters. We used a Bayesian analysis employing Markov chain Monte Carlo (MCMC) simulation for the posterior $p(g_i,\tau_i,\theta_i|F) \propto p(\theta_i)p(g_i)p(\tau_i)L_i$, (for MCMC techniques see Gilks et al. Markov chain Monte Carlo in Practice, Chapman & Hall, 1995). By using MCMC we can simulate the joint posterior on the parameters, and thus examine correlations and calculate any moments of interest. Computation of the posterior requires priors to be given for the parameters. We used a uniform prior for g_i on interval [0,3], a uniform prior on [0,1] for q. We use a conjugate prior for the random walk in the width precision parametrised by d, $p(d) = \Gamma(a,b)$ with a, b chosen so that the prior is weak. We used random walk proposals on all variables, $v' \sim N(v,\sigma_v^2)$, the variance σ_v^2 being tuned during burn-in to give an acceptance probability between 0.2-0.4. The efficiency of the algorithm can be improved by reparametrisation.

4 Effect of the point spread function on exclusion energy correlation.

Here we examine the effect of the point spread function (PSF) on our inferred g_1, g_2 diagram. We use the protein-rich supported bilayer experimental data to illustrate its effects; the linearity in the NK synapse after deconvolution being much stronger so the effect of the PSF is less of an issue, whilst the 2D data, with the higher ligand levels and thus lower stochasticity/level of fluctuations has weaker signals as discussed below.

Firstly, we compare raw and deconvolved experimental data, Fig. S3. These give similar results, specifically the linear relation is apparent in both. However, there are distinct differences: the background distribution is wider after deconvolution, with relative standard deviations of (red channel) 9% and 17% respectively. This increase in the uncertainty of the microscope properties,

(the background infers $\alpha_i, \mu_i L_i^{fs}$), explains the increase in the transverse spread in the g_1, g_2 plot which is the major cause of the degraded linearity. Of more significance is the emergence of synapse pixels with CD58 fluorescence below the background in the deconvolved image. These are both a consequence of the fact that the PSF averages intensities. Thus, for instance, the heterogeneities in the bilayer (which exclude both fluorphores) cause a spread in background intensity, that under PSF averaging are less apparent, whilst CD58 intensity in the interface has a reduced range because of local averaging over the pattern removing extreme values. Since deconvolution is inexact, we also examine a resized PSF by a factor of 1.2, Figs. S3I-L. Results are again similar suggesting that the linear relationship is robust to the effects of the PSF. However, there are quantitative effects on the gradient: under joint channel inference we obtain a gradient -0.406 ± 0.003 (not deconvolved), -0.46 ± 0.005 (deconvolved). Clearly the inference could be improved by also modelling the heterogeneity of the bilayer since it is a source of variability.

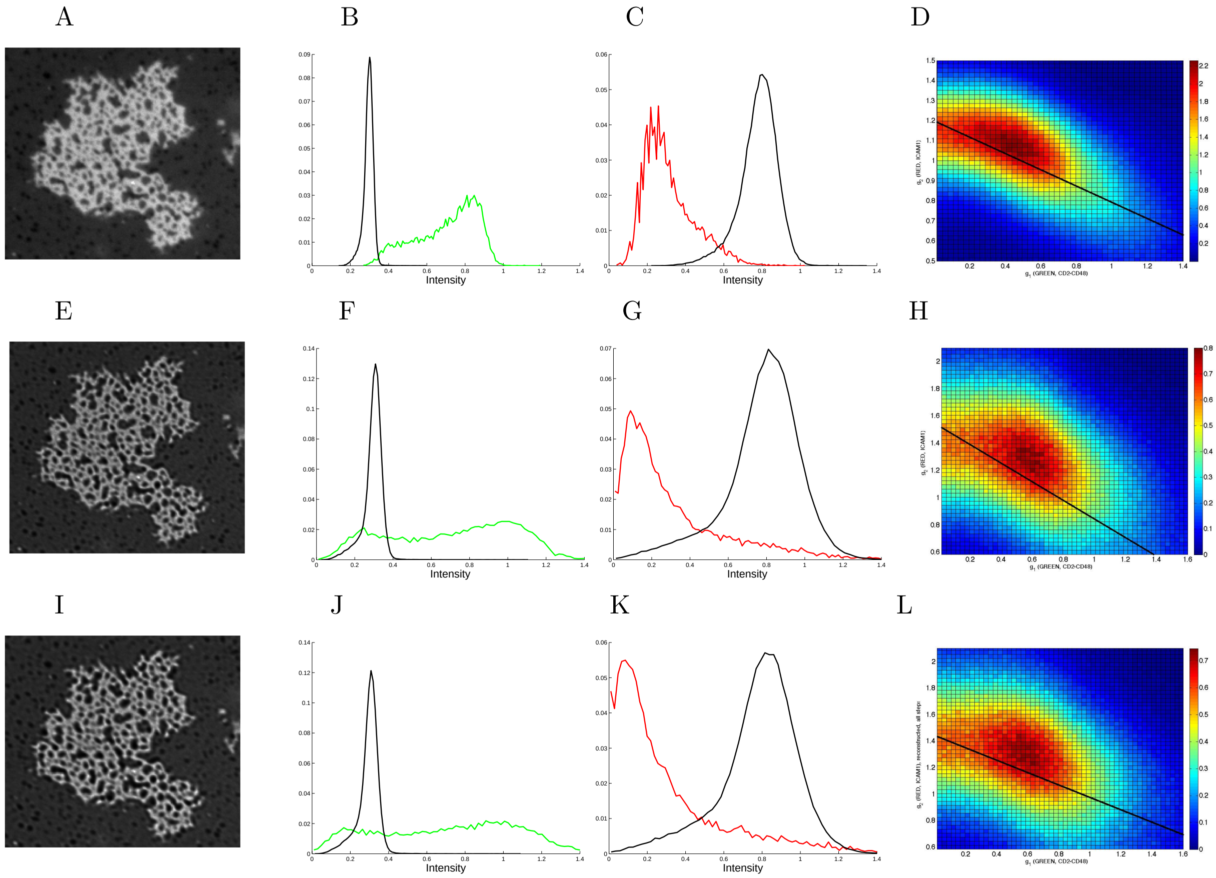

Figure 3. Analysis of bilayer experimental data (Fig. 1) comparing processed, but not deconvolved data, with deconvolved data. **A/E/I**. CD58 image. **B/F/J**. Intensity histograms CD58 (green, background black). **C/G/K**. Intensity histogram ICAM1 (red, background black). **D/H/L**. Inferred g_1, g_2 diagram. First row (A-D), processed but not deconvolved data, second row (E-H), deconvolved, third row (I-L), deconvolved with enlarged PSF (20%). The g_1, g_2 plot is based on separate channel reconstructions, and the (mean) regression line inferred from joint analysis is shown (black). The PSF was estimated by imaging fluorescent beads.

5 Step potential model and PSF linearity

Our ability to detect a linear relationship between g_1, g_2 caused by size exclusion relies on intermediate membrane separations occuring in the image, *i.e.* intermediate fluorescence between maximal enrichment (exclusion) and the background for CD58 (ICAM1 respectively). If the domain walls are sub-light resolution and are homogeneous in their concentrations then this will not be the case. Higher receptor/ligand concentrations reduce the level of fluctuations and thus the domain walls will be sharper. This step potential model (with step changes in concentrations at domains) can be analysed in considerable detail and aids understanding of the effect of the PSF. Further, we can ask to what degree the PSF alone can produce g_1, g_2 patterns similar to those observed.

A B

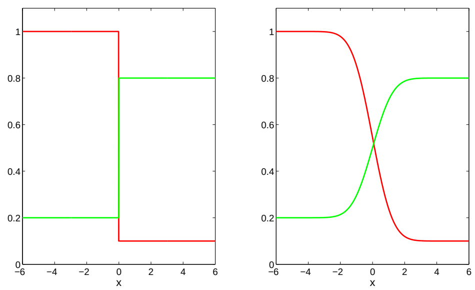

Figure 4. Schematic illustrating the effect of the PSF on fluorescence under a discrete valued potential (concentration) model. A single domain wall at $x = 0$ is illustrated, **A.** intensity of red and green fluorophores in absence of PSF, and **B** after PSF applied.

Here, we examine this Boolean (step) model, simulating the observed patterns for comparison to the undeconvolved case in Fig. S3. Thus, domains are modelled as 'tight' entities with uniform complex and ICAM1 concentrations, ie within the synapse there are only two concentration states, high ICAM1 (low CD58) and low ICAM1 (high CD58) with domain walls assumed sub-light microscopy resolution. Thus, intermediate fluorescence values arise through effects of the PSF alone, Fig. S4. We simulate this using one of the synapse patterns observed in the bilayer experiments and the measured PSF. By choosing the minimum and maximum concentrations of ICAM1 and CD58 respectively, and optionally adding fluorescence noise (to match the free surface fluorescence distribution), we can obtain an approximate match to the synapse fluorescence distribution. The simulated fluorescence prior to PSF convolution (Fig. S5A) should be compared to the deconvolved data of Fig. S3E, and the convolved simulation (Fig. S5B) with the original experimental image data, Fig. S3A. As is clear, the PSF produces a small region of approximate linearity with gradient -0.466±0.005, close to that inferred on the original image. However, the corresponding g_1, g_2 patterns Fig. S5C, S3H, and S5D, S3D show distinct differences.

A B C D

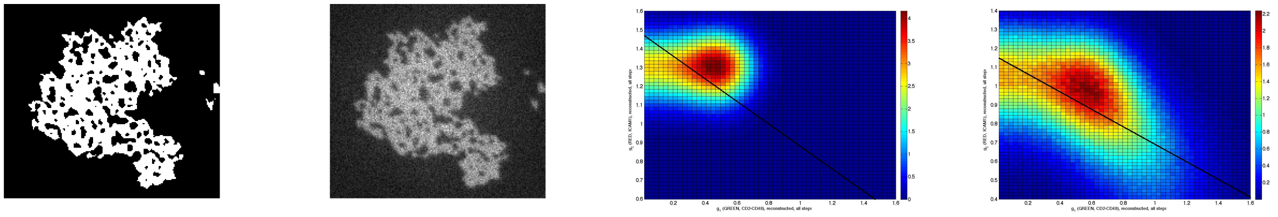

Figure 5. Simulated image data and inferred g_1, g_2 plot for the step concentration model with PSF. **A**. Thresholded image (CD58), white regions are regions of enriched CD58. **B**. Simulated CD58 under PSF and added noise, **C**. Inferred g_1, g_2 diagram for thresholded image (with added noise). **D**. Inferred g_1, g_2 diagram for simulated image under PSF.

The source of this linearity can be analysed analytically, and it's dependencies determined.

Firstly, we simplify the problem, analysing a 1D domain with a wall at $x = 0$, Fig. S4 and define the two concentration states as (C_{max}, B_{min}) and (C_{min}, B_{max}). We have (mean) fluorescence, green and red channels with PSFs $\Psi_{g/r}$ respectively, at a distance x from the interface given by,

$$\begin{aligned} G(x) &= \alpha_1\mu_1 C_{max}e^{-g_1^2} = \alpha_1\mu_1(C_{max} - C_{min})\int_{-\infty}^{0}\Psi_g(x-y)dy + \alpha_1\mu_1 C_{min}, \\ R(x) &= \alpha_2\mu_2 B_{max}e^{-g_2^2} = \alpha_2\mu_2(B_{max} - B_{min})\int_{0}^{\infty}\Psi_r(x-y)dy + \alpha_2\mu_2 B_{min} \end{aligned} \tag{8}$$

Here we are only modelling the fluorescence from the complex; thus fluorescence from the unbound ligand needs to be added.

For simplicity, assume that the PSFs for the red and green channels are identical (we readdress this below). Then we deduce that the following relation holds,

$$\frac{G(x)}{C_{max} - C_{min}} + \frac{R(x)}{B_{max} - B_{min}} = h = 1 + \frac{C_{min}}{C_{max} - C_{min}} + \frac{B_{min}}{B_{max} - B_{min}} \tag{9}$$

since $\int_{-\infty}^{\infty}\Psi(x-y)dy = 1$. The required relation between g_1, g_2 now follows,

$$\frac{\exp -g_1(x)^2}{1 - C_{min}/C_{max}} + \frac{\exp -g_2(x)^2}{1 - B_{min}/B_{max}} = h. \tag{10}$$

Note that if $g_1 = 0$ then $e^{-g_2^2} = B_{min}/B_{max}$ as expected. Fluorescence, under a PSF, thus tracks this curve, Fig. S6. Although derived for 1D, this analysis applies to both 2 and 3D since the domains are sufficiently large relative to the PSF width that domain walls appear 1 dimensional.

We can now estimate the gradient. Since the synapse is dominated by the state with low ICAM, this point dominates the 2D histogram, as seen in Fig. S3. The gradient is given by,

$$\frac{dg_2}{dg_1} = -\frac{g_1}{g_2}e^{-g_1^2+g_2^2}\frac{1 - B_{min}/B_{max}}{1 - C_{min}/C_{max}} \tag{11}$$

Typically $C_{min} \ll C_{max}$, while $B_{min}/B_{max} \approx 0.2$.

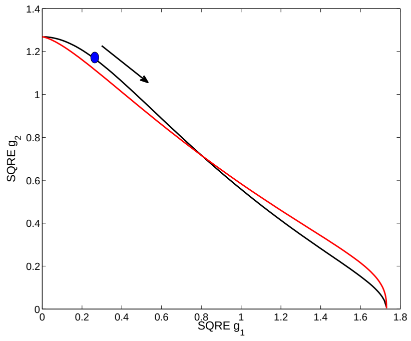

Figure 6. PSF incurred correlation patterns on the exclusion energy diagram g_1, g_2. Black, identical PSF for red and green channels, Eqn. (S10). Red, 20% increase in red PSF Gaussian width, Eqn. (S12). Blue dot denotes dominant state and arrows the shift of pixel exclusion energies under the PSF.

The effect of the PSF being wavelength dependent can also be examined. However, to do this we need a functional form for the PSF. For simplicity we use a Gaussian, which is a good approximation for the main peak of the Airy function. The relation Eqn. (S8) is now an error function,

$$\begin{aligned} G(x) &= C_{max}e^{-g_1^2} = \frac{1}{2}(C_{max} - C_{min})erfc(\frac{x}{\sqrt(2)\sigma_g}) + C_{min}, \\ R(x) &= B_{max}e^{-g_2^2} = (B_{max} - B_{min})\left(1 - \frac{1}{2}erfc(-\frac{x}{\sqrt(2)\sigma_r})\right) + B_{min} \end{aligned} \tag{12}$$

Thus, we can map out the $g_1 g_2$ curve as a parametrised curve in x, Fig. S6. This demonstrates that the functional form is continuously modified, *i.e.* there is structural stability. Thus, the 8% difference in PSF widths will perturb the gradient only marginally. The effect is therefore negligible.

Finally, we can determine the fluorescence redistribution under this model, ie the degree of shift of the fluorescence population along the contour of Fig. S6 due to the effects of the PSF. If $P(x)$ is the distribution of distances of pixels from a domain, we have $P(G)dG = P(x)dx$, which, using Eqn (S11) gives the distribution $P(G)$. The distribution $P(x)$ is empirically L_x/A where L_x is the length of the contours at a distance x from the closest domain. If the domain is circular of radius r, $L_x = (1 + \frac{x}{r})L_0$. Domains are not isolated, which will decrease L_x at $x > r$, whilst domains are not circular, or of constant size, suggesting $P(x)$ will be roughly Gaussian with mean, sd on the scale of the natural spatial scale of the domain structure (size, separation distance). This can be calculated for the data in Fig. S5A.

Our analysis indicates that the PSF can produce a linear relation between g_1, g_2, Fig. S6, the gradient being determined primarily from the position of the main fluorescence population, Eqn. (S11). Qualitatively the patterns are similar, original image (not deconvolved) Fig. S3D and reconstruction under step concentration model Fig. S5D, although the spread along the line of regression is less pronounced in the latter. However, as demonstrated above, linearity between g_1, g_2 remains after deconvolution, Fig. S3H, and distinctly different than the step model pattern, Fig. S5C. Thus, although the PSF produces a linear relation between g_1, g_2, our analysis indicates that it is not the sole source of this linearity.

Acknowledgements. MatLab was used throughout for analysis and image database management.
